# Supplementary figures and images for: A multi-method approach for assessing the distribution of a rare, burrowing North American crayfish species
Source: PeerJ. 2023 Feb 20;11:e14748. doi: 10.7717/peerj.14748 (PMC9948742; doi:10.7717/peerj.14748)

HUC 8 Watersheds

**Precision**

- Exact
- Good
- Fair
- Poor

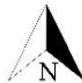

0 25 50 100 Kilometers

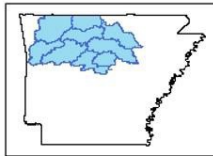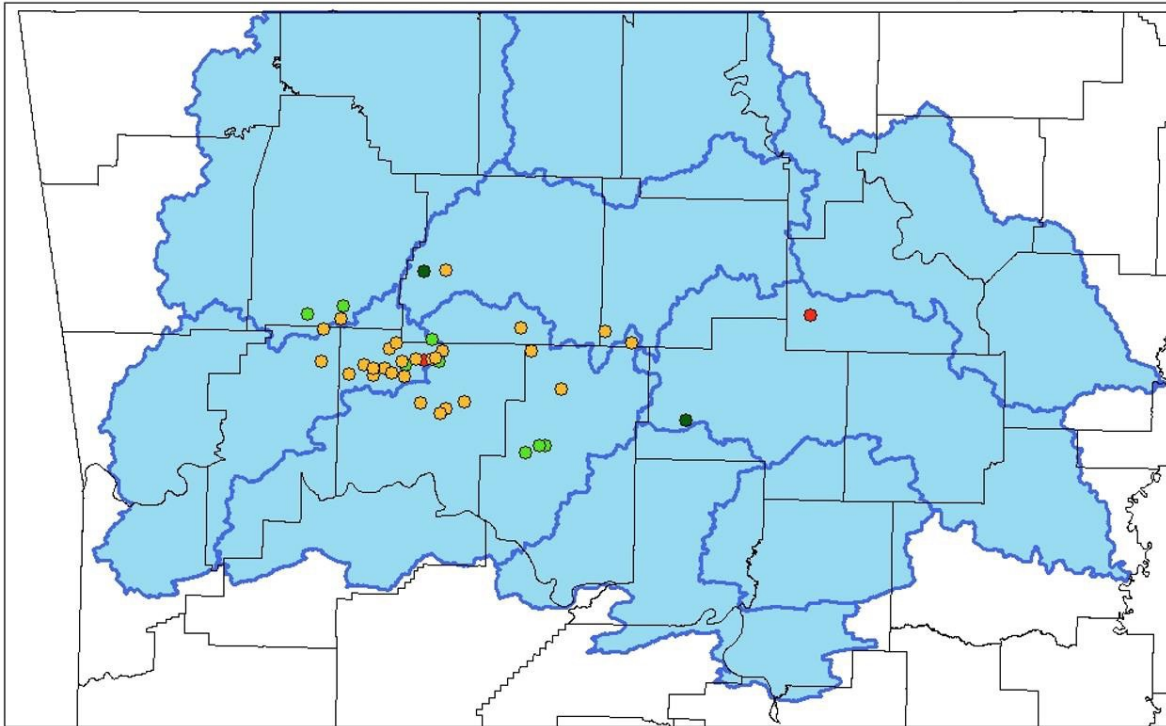

Supplement: Figure S1 — A map of Arkansas, USA showing the extent of the MaxEnt model for Cambarus causeyi, all historical sites, confidence in site accuracies, and the HUC8’s that make up the extent. “Poor” sites were not used in MaxEnt model, and “unusable” sites are absent from this map because they could not be georeferenced. HUC8 watersheds visualized in the inset map were used as the study extent because they overlapped with counties (black lines) with historical records. [file peerj-11-14748-s001.pdf]

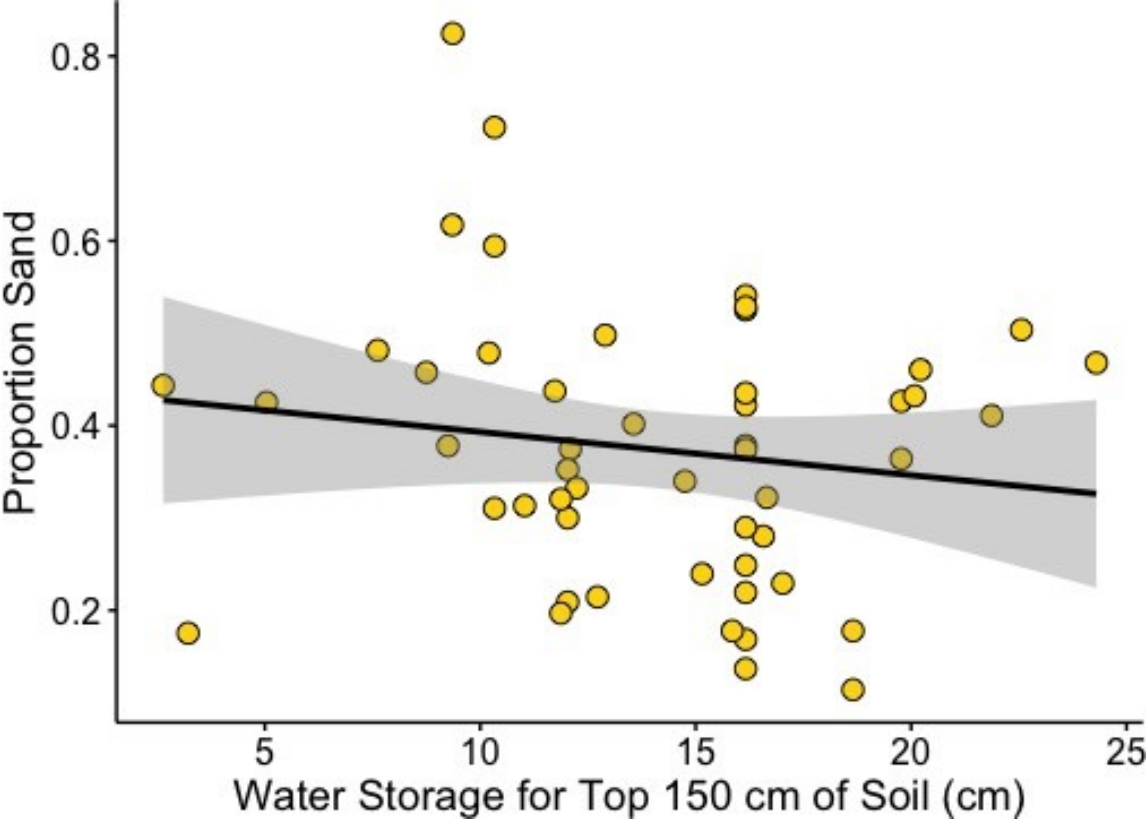

Supplement: Figure S2 — Linear regression between proportion of sand in soil from study sites and estimated water storage capacity from gSSURGO with 95% confidence intervals (R2 = 0.021, p = 0.31). [file peerj-11-14748-s002.pdf]
