# Supplementary material for: A multi-method approach for assessing the distribution of a rare, burrowing North American crayfish species
Source: PeerJ. 2023 Feb 20;11:e14748. doi: 10.7717/peerj.14748 (PMC9948742; doi:10.7717/peerj.14748)
Supplement: Table S1 — Soil texture data collected during Cambarus causeyi field sampling and analyzed using laser diffraction at the Illinois State Water Survey. [file peerj-11-14748-s003.docx]

| **Field #** | **Date** | **Latitude** | **Longitude** | **County** | **%**  **Sand** | **%**  **Silt** | **%**  **Clay**  **<2µm** | **USDA**  **Class** |
| --- | --- | --- | --- | --- | --- | --- | --- | --- |
| KBQ  19-16 | 2019-  05-21 | 35.62994 | -93.74456 | Franklin | 22.93 | 63.65 | 13.42 | ZL |
| KBQ  19-17 | 2019-  05-21 | 35.67702 | -93.73857 | Franklin | 28.03 | 59.21 | 12.76 | ZL |
| KBQ  19-18 | 2019-  05-21 | 35.83255 | -93.75476 | Madison | 36.41 | 54.91 | 8.68 | ZL |
| KBQ  19-20 | 2019-  05-21 | 35.76342 | -93.74209 | Franklin | 17.78 | 69.80 | 12.42 | ZL |
| KBQ  19-21 | 2019-  05-22 | 35.67571 | -93.56564 | Johnson | 37.84 | 55.48 | 6.68 | ZL |
| KBQ  19-22 | 2019-  05-22 | 35.67506 | -93.56461 | Johnson | 52.53 | 42.10 | 5.37 | SL |
| KBQ  19-23 | 2019-  05-22 | 35.68594 | -93.453 | Johnson | 41.08 | 54.73 | 4.19 | ZL |
| KBQ  19-24 | 2019-  05-22 | 35.75005 | -93.45097 | Newton | 49.79 | 45.13 | 5.08 | SL |
| KBQ  19-25 | 2019-  05-22 | 35.59256 | -93.41238 | Johnson | 24.88 | 59.33 | 15.79 | ZL |
| KBQ  19-26 | 2019-  05-23 | 35.58891 | -93.34202 | Johnson | 16.85 | 74.57 | 8.58 | ZL |
| KBQ  19-27 | 2019-  05-23 | 35.70903 | -93.1517 | Pope | 21.94 | 61.72 | 16.35 | ZL |
| KBQ  19-30 | 2019-  05-23 | 35.58723 | -93.06491 | Pope | 21.45 | 62.57 | 15.98 | ZL |
| KBQ  19-32 | 2019-  05-23 | 35.49232 | -93.12066 | Pope | 32.22 | 61.65 | 6.13 | ZL |
| KBQ  19-34 | 2019-  05-24 | 35.58287 | -93.46875 | Johnson | 37.47 | 58.21 | 4.32 | ZL |
| KBQ  19-36 | 2019-  06-04 | 35.68678 | -94.07263 | Crawford | 37.42 | 58.01 | 4.57 | ZL |
| KBQ  19-37 | 2019-  06-04 | 35.69694 | -94.04698 | Crawford | 61.72 | 34.16 | 4.12 | SL |
| KBQ 19-38 | 2019-  06-04 | 35.73243 | -94.04498 | Crawford | 54.07 | 43.79 | 2.14 | SL |
| KBQ  19-39 | 2019-  06-04 | 35.73484 | -94.07069 | Crawford | 42.21 | 49.71 | 8.07 | L |

| KBQ  19-40 | 2019-  06-04 | 35.76623 | -93.91526 | Madison | 47.86 | 46.49 | 5.65 | SL |
| --- | --- | --- | --- | --- | --- | --- | --- | --- |
| KBQ  19-41 | 2019-  06-05 | 35.89798 | -93.45442 | Newton | 35.25 | 59.97 | 4.78 | ZL |
| KBQ  19-42 | 2019-  06-05 | 35.94535 | -93.42053 | Newton | 20.90 | 62.58 | 16.52 | ZL |
| KBQ  19-43 | 2019-  06-05 | 35.89432 | -93.39811 | Newton | 28.98 | 53.75 | 17.27 | ZL |
| KBQ 19-45 | 2019-  06-05 | 35.72516 | -92.94542 | Searcy | 48.15 | 46.71 | 5.14 | SL |
| KBQ  19-46 | 2019-  06-05 | 35.73959 | -92.93436 | Searcy | 33.24 | 58.73 | 8.03 | ZL |
| KBQ  19-47 | 2019-  06-06 | 35.55 | -92.70353 | Van Buren | 40.18 | 52.60 | 7.22 | ZL |
| KBQ  19-51 | 2019-  06-06 | 35.54151 | -92.69925 | Van Buren | 17.78 | 71.94 | 10.28 | ZL |
| KBQ  19-52 | 2019-  06-06 | 35.79359 | -92.3409 | Stone | 82.45 | 15.94 | 1.60 | LS |
| KBQ  20-74 | 2020-  06-02 | 35.81398 | -94.10864 | Washington | 42.62 | 49.27 | 8.11 | L |
| KBQ  20-75 | 2020-  06-03 | 35.67097 | -93.80508 | Franklin | 43.75 | 48.92 | 7.33 | L |
| KBQ 20-76 | 2020-  06-10 | 35.74936 | -92.89838 | Searcy | 31.33 | 57.05 | 11.62 | ZL |
| KBQ  20-77 | 2020-  06-09 | 35.82126 | -93.35729 | Newton | 43.53 | 49.54 | 6.93 | SL |
| KBQ  20-78 | 2020-  06-09 | 35.84814 | -93.19154 | Newton | 30 | 59.18 | 10.82 | ZL |
| KBQ  20-79 | 2020-  06-10 | 35.72161 | -92.84055 | Pope | 32.04 | 63.12 | 4.84 | ZL |
| KBQ  20-80 | 2020-  06-02 | 35.94375  122 | -93.974275 | Washington | 46.79 | 51 | 2.2 | ZL |
| KBQ  20-81 | 2020-  06-02 | 35.84113 | -93.8818 | Madison | 23.96 | 67 | 9.04 | ZL |
| KBQ  20-82 | 2020-  06-02 | 35.854 | -94.00474 | Washington | 33.99 | 56.16 | 9.85 | ZL |
| KBQ  20-83 | 2020-  06-03 | 35.69476 | -93.97803 | Franklin | 37.81 | 53.51 | 8.67 | ZL |
| KBQ  20-84 | 2020-  06-03 | 35.55908 | -93.98834 | Franklin | 17.53 | 72.19 | 10.28 | ZL |
| KBQ  20-85 | 2020-  06-03 | 35.53491 | -93.53657 | Johnson | 43.22 | 51.89 | 4.9 | ZL |
| KBQ  20-86 | 2020-  06-04 | 35.50522 | -93.9748 | Franklin | 42.51 | 52.01 | 5.48 | ZL |

| KBQ  20-87 | 2020-  06-04 | 35.68840  4 | -93.238246 | Johnson | 44.35 | 48.56 | 7.09 | L |
| --- | --- | --- | --- | --- | --- | --- | --- | --- |
| KBQ  20-88 | 2020-  06-04 | 35.57977 | -93.14053 | Pope | 19.69 | 74.21 | 6.1 | ZL |
| KBQ  20-89 | 2020-  06-04 | 35.59046 | -92.95174 | Pope | 46.05 | 47.67 | 6.28 | SL |
| KBQ  20-90 | 2020-  06-09 | 35.82127 | -93.35725 | Newton | 52.86 | 44.88 | 2.26 | SL |
| KBQ 20-91 | 2020-  06-09 | 35.82127 | -93.35725 | Newton | 13.68 | 60.7 | 25.62 | ZL |
| KBQ  20-92 | 2020-  06-09 | 35.77106 | -93.159 | Newton | 50.38 | 45.27 | 4.35 | SL |
| KBQ  20-93 | 2020-  06-10 | 35.7537 | -93.0954 | Newton | 11.41 | 70.69 | 17.9 | ZL |
| KBQ  20-94 | 2020-  06-11 | 35.68989 | -92.6584 | Van Buren | 45.76 | 50.33 | 3.9 | ZL |
| KBQ  20-95 | 2020-  06-11 | 35.7737 | -92.29342 | Stone | 72.29 | 24.28 | 3.43 | SL |
| KBQ  20-96 | 2020-  06-11 | 35.78939 | -92.30533 | Stone | 59.45 | 38.52 | 2.03 | SL |
| KBQ  20-97 | 2020-  06-11 | 35.79129 | -92.30531 | Stone | 31.05 | 59.71 | 9.24 | ZL |
