# Supplementary material for: A multi-method approach for assessing the distribution of a rare, burrowing North American crayfish species
Source: PeerJ. 2023 Feb 20;11:e14748. doi: 10.7717/peerj.14748 (PMC9948742; doi:10.7717/peerj.14748)
Supplement: Table S2 — The gBlock sequences of Cambarus causeyi used to develop the primer-probe assay and the non-target crayfish species that the C. causeyi assay was tested in silico and in vitro. [file peerj-11-14748-s004.docx]

| **Non-target species** | **GenBank Accession** | **gBlock sequence** |
| --- | --- | --- |
| *Cambarus causeyi* | JX514477 | TATTAACATGCGTGCGGCGGGTATAACTATAGATCGTATACCTTTGTTTGTTTGGTCTGTATTTGTTACTACTGTTTTACTATTGTTATCTTTGCCTGTGCTAGCGGGGGCTATTACTATGCTATTAACAGATCGTAATTTAAATACTTCT |
| *Cambarus hubbsi* | MG872957 | AATATGCGAATAGTAGGTATAACTATGGATCGCATGCCTTTATTTGTTTGGTCCGTGTTTATTACTACTGTTTTATTATTATTATCTTTACCTGTGTTAGCAGGAGCAATCACTATGTTGTTGACGGATCGGAATTTA |
| *Procambarus liberorum* | KF827978 | AATATACGAACGGTAGGGATAACTATAGATCGAATACCATTATTTGTTTGATCTGTATTTATTACTACTGTACTATTGTTGTTATCTCTACCTGTATTGGCAGGGGCTATTACTATACTATTAACAGACCGGAATCTA |
| *Faxonius williamsi* | AY701252 | AACATGCGTTCAGCTGGAATAACTATGGATCGGATACCGTTATTTGTTTGATCAGTATTTATTACTACTGTGTTATTGTTATTATCTTTACCTGTTTTAGCAGGGGCAATCACTATATTATTAACTGATCGAAATTTA |
| *Faxonius meeki meeki* | AY701213.1 | AATATACGGGCTGTAGGTATAACTATGGATCGTATACCGTTATTTGTTTGATCAGTGTTTATTACTACTGTGTTATTATTGTTATCTTTACCTGTTTTGGCTGGGGCAATTACTATATTATTAACTGATCGTAATTTA |
